# Recent advances in the application of ionomics in metabolic diseases

**DOI:** 10.3389/fnut.2022.1111933

**Published:** 2023-01-16

**Authors:** Yan Zhang, Biyan Huang, Jiao Jin, Yao Xiao, Huimin Ying

**Affiliations:** ^1^Shenzhen Key Laboratory of Marine Bioresources and Ecology, Brain Disease and Big Data Research Institute, College of Life Sciences and Oceanography, Shenzhen University, Shenzhen, China; ^2^Shenzhen-Hong Kong Institute of Brain Science-Shenzhen Fundamental Research Institutions, Shenzhen, China; ^3^Affiliated Hangzhou Xixi Hospital, Zhejiang University School of Medicine, Hangzhou, China

**Keywords:** trace element, ionome, ionomics, metabolic disease, bioinformatics

## Abstract

Trace elements and minerals play a significant role in human health and diseases. In recent years, ionomics has been rapidly and widely applied to explore the distribution, regulation, and crosstalk of different elements in various physiological and pathological processes. On the basis of multi-elemental analytical techniques and bioinformatics methods, it is possible to elucidate the relationship between the metabolism and homeostasis of diverse elements and common diseases. The current review aims to provide an overview of recent advances in the application of ionomics in metabolic disease research. We mainly focuses on the studies about ionomic or multi-elemental profiling of different biological samples for several major types of metabolic diseases, such as diabetes mellitus, obesity, and metabolic syndrome, which reveal distinct and dynamic patterns of ion contents and their potential benefits in the detection and prognosis of these illnesses. Accumulation of copper, selenium, and environmental toxic metals as well as deficiency of zinc and magnesium appear to be the most significant risk factors for the majority of metabolic diseases, suggesting that imbalance of these elements may be involved in the pathogenesis of these diseases. Moreover, each type of metabolic diseases has shown a relatively unique distribution of ions in biofluids and hair/nails from patients, which might serve as potential indicators for the respective disease. Overall, ionomics not only improves our understanding of the association between elemental dyshomeostasis and the development of metabolic disease but also assists in the identification of new potential diagnostic and prognostic markers in translational medicine.

## 1. Introduction

Metabolic diseases are well-known as a major threat to global public health, which include diabetes mellitus (DM), obesity, metabolic syndrome (MetS), non-alcoholic fatty liver disease (NAFLD), hyperlipidemia, hyperuricemia (or gout), bone metabolic disease (such as osteoporosis and osteomalacia), and some other diseases that are caused by endocrine dysfunction (e.g., thyroid diseases) or nutrient imbalance (such as Wilson's disease) (1, 2). In the past several decades, much effort has been made to understand the underlying mechanisms and to develop rational treatments for these diseases and disorders. However, due to the complex pathogenesis and characteristics of most metabolic diseases, the progress still remains quite limited. Thus, effective strategies of early diagnosis and treatment of metabolic diseases are urgently needed.

Among known risk factors and possible molecular mechanisms associated with metabolic diseases, dyshomeostasis of various elements has been implicated in the onset and progression of many types of them (3–6). Except for a small number of elements that are required in large amounts (called macroelements or macronutrients), such as potassium (K), sodium (Na), calcium (Ca), and magnesium (Mg), the majority of them are trace elements. Biological trace elements (or micronutrients, mostly metals) are needed in small quantities but are essential for growth, development, and various physiological processes of all living organisms, which include iron (Fe), zinc (Zn), copper (Cu), manganese (Mn), molybdenum (Mo), nickel (Ni), cobalt (Co), chromium (Cr), vanadium (V), selenium (Se), iodine (I), and several other elements (7, 8). They serve as critical cofactors for the assembly of metalloproteins, which participate in a very wide range of biological processes such as enzymatic reaction, cellular signaling transduction, redox regulation, mitochondrial function, immunological response, and hormone and vitamin biosynthesis (9, 10). In addition, several environmental toxic elements, such as cadmium (Cd), mercury (Hg), arsenic (As), lead (Pb), and aluminum (Al), have detrimental effects on human health (11, 12).

It has been known that the cell has developed complex regulatory mechanisms (such as uptake, excretion, and storage) to tightly control the metabolism and homeostasis of various trace elements in order to ensure appropriate supply while preventing accumulation to toxic levels (13). Previously, a large number of studies have reported that deficiency or overload of certain trace elements are related to a variety of metabolic diseases and disorders (14–18). For example, Fe deficiency is significantly associated with obesity, whereas excessive Fe accumulation in adipose tissue and liver is often seen in type 2 diabetes mellitus (T2DM) and NAFLD (14, 15). Furthermore, some elements may interact and interfere with the absorption, bioavailability, and even normal functions of others (19–21). On the other hand, trace element levels may be altered in various clinical conditions, which may be associated with the prognosis and efficacy of the treatment (22). Although numerous studies have been performed to evaluate the status of single or several elements in biological samples of different types of metabolic diseases, it is still unclear how disruption of the balance of various elements are involved in the development and progression of these diseases. A more systematic understanding of the metabolism and homeostasis of trace elements and their relationships with metabolic diseases is urgently needed.

In the recent 20 years, with the explosive growth of multi-omics data (such as genome, transcriptome, and proteome) and a corresponding increase in data analysis methods, the complex relationship between trace elements and human health or diseases has been examined comprehensively. The concept of metallome (the ensemble of all metal ions in an organism) and its extension ionome (representing the composition of all metals, metalloids, and non-metals found in a living system) have also been introduced (23, 24). Ionomics, the study of the ionome, involves simultaneous and quantitative analyses of elemental composition in living systems (such as cells, tissues, and organs) and changes in this composition under different physiological and pathological conditions by using high-throughput elemental profiling techniques (24–26). This approach has been extensively applied in plants, yeast, and most recently mammals (including humans), which provides a very useful platform for not only identifying new characteristics and components of trace element metabolism and homeostasis but also gaining information about how the complex ionomic network is affected by different factors and states (26–28). In addition, isotopic and speciation analysis of elements is becoming an inherent or complementary part of ionomics, which is important for a more accurate assessment of the status of elements present in biological samples (29, 30). In recent years, ionomics approach has been applied to study the global effect of a large amount of elements for various human diseases, which offers a great opportunity to discover new ion-based mechanisms of these diseases (especially complex disease) and to enable the possibility to develop new diagnostic tools and therapeutic strategies (31, 32).

In this review, we present a comprehensive survey on the complex relationship between ionomic profiles in diverse human biological samples and several major types of metabolic diseases mainly based on recent large-scale ionomic/metallomic studies, such as case-control studies, cohort- and population-based studies, and meta-analyses. Such information may provide a holistic and integrated understanding of the critical roles trace elements play in metabolic diseases.

## 2. Ionomics technology and its application in human health and diseases

The major purpose of ionomics is to measure the ion contents in a biological system and to elucidate their correlations to phenotypes of interest. Nowadays ionomics has been widely used for studies on environmental monitoring, nutrient utilization, biofortification, food safety, and forward and reverse genetics, particularly in plant developmental and stress biology which has been extensively reviewed in the literature (23–25, 33–35). In addition, the rapid expansion of ionomics in human health and diseases has provided new evidence for the relationship between trace elements and a variety of common diseases, which may be helpful for the early detection of disease and the innovation of new drugs against imbalanced elements.

### 2.1. Techniques for elemental quantification and related data resources

In early times, elemental profiling analysis in various samples was mainly used in geoscience, environmental science, and ecological studies. In the last decade, ionomics has grown in popularity in different areas of life science as a powerful tool for studying the metabolism and homeostasis of diverse trace elements and minerals in different organisms, including plants and humans. The major techniques for the quantification of chemical elements include inductively coupled plasma mass spectrometry (ICP-MS), inductively coupled plasma atomic/optical emission spectroscopy (ICP-AES/ICP-OES), X-ray fluorescence (XRF), atomic absorption spectrometry (AAS), neutron activation analysis (NAA), and several other methods (23, 33). Currently, ICP-MS and ICP-AES are the two most widely used high-throughput analytical tools. ICP-MS has been the dominant technique for the analysis of multi-elemental concentrations due to its high accuracy, high sensitivity, wide measurement capability, and the ability for isotopic analysis (36). Although ICP-AES/ICP-OES is less sensitive than ICP-MS, it offers the advantages of lower cost and simplicity of operation (37). Both methods have been used for ionomics studies in yeast, plants, and animals, which demonstrate the potential benefits of ionomics in the exploration of new aspects of trace elements in biology and medicine (26, 27, 38–40). Other techniques, although used less frequently, may provide alternative approaches for determining various elements from different samples (41, 42).

With the increase in volume of data generated by ionomics studies, it becomes more and more important to develop appropriate information management systems for ionomics data acquisition, storage, retrieval, and analysis. However, publicly accessible platforms and databases that allow researchers to obtain such resources are very rare so far. The iHUB system is the first integrated workflow system, which contains ionomics data of yeast and important plants such as Arabidopsis thaliana, maize, rice, and soybean, and provides open access for raw data management, data mining and analysis, and knowledge discovery (43). The OPTIMAS Data Warehouse (OPTIMAS-DW) is a comprehensive and integrated data resource for maize, which contains ionomics and other omics data such as transcriptome, proteome, metabolome, and phenome, and allows users to extract information of particular interest (44). Unfortunately, an ionomics database associated with human disease is still absent.

On the other hand, with the rapid progress in the experimental and computational identification of novel trace element-related genes and their functions, relevant information should be well-maintained for further utilization. A known ionome gene (KIG) database was recently developed, which is a curated list of genes known to affect uptake, accumulation, and distribution of elements in several plant species (45). New systems and annotation tools are clearly required to obtain a better understanding of the function and regulation of genes affecting the ionome on a genome-wide scale.

### 2.2. Bioinformatics for ionomics data analysis

The recent explosion of ionomics data has prompted the development of computational strategies and methods for investigation of the associations between ionomic profiles and complex traits. Bioinformatics approaches for ionomics data analysis include both basic statistical methods (such as statistical significance testing and analysis of variance) and advanced multivariate statistical methods (e.g., principal component analysis and logistic regression analysis), which could also be applied for other types of omics data (31). Moreover, network-based systems biology approaches and machine learning algorithms have been used to clarify the dynamic interplay between elements under different physiological or metabolic conditions and to build ionome-based prediction models or markers for a variety of diseases (46–49). Very recently, an integrated R-based tool called IonFlow was developed for the analysis of ionomics data sets, which may help researchers to process, explore, and interpret their data *via* multiple approaches (50).

### 2.3. Application of ionomics in human disease

Before the concept of ionomics was introduced, ICP-MS had been used to evaluate the concentrations of certain trace elements in biofluids, hair, nails, and some other human tissues for different diseases such as cancer, neurodegenerative diseases, and coronary heart disease, demonstrating that imbalance of trace elements may be one of the risk factors of many complex or common diseases (51–53). Most of these preliminary studies only focused on single or a very limited number of trace elements and lacked deep analysis of the interactions between elements. In recent years, ionome-based studies have been performed for a variety of complex diseases, which may assist in diagnosis and prognosis evaluation of disease. Considering that most ionomics results have been derived from cross-sectional or case-control studies, it is uncertain whether disrupted ion homeostasis is a potential cause of these diseases. Large-scale prospective, longitudinal, and interventional studies are necessary to solve these questions. In addition, the intrinsic limitation of current elemental analytical techniques and the validity and reliability of using ionomic information in the prediction of diseases imply that there is still a long way ahead of us before ionomics can be widely used in medical clinics. However, the application of ionomics in several types of diseases has provided evidence for its potential benefits, which suggests significant correlations between trace elements and the development of different diseases and may help early detection and improve therapeutic regimens for patients in the future.

## 3. Recent progress in ionomics of metabolic diseases

It has been known for a long time that trace elements and minerals play a critical role in the onset and progression of metabolic diseases (32, 54, 55). Previously, lots of studies have been conducted to explore the linkages between single or multiple elements and various metabolic diseases, such as the association between intake of certain elements and the incidence of DM and osteoporosis and the implication of heavy metal exposure in the epidemiology of metabolic diseases (56–58). In recent years, ionomics approaches have been used to examine the variations of elemental profiles in different body fluids and tissues from patients with metabolic diseases, providing new clues for not only better understanding of the relationship between trace element dyshomeostasis and these diseases but also the development of novel biomarkers for detection, diagnosis, and prognosis of them. In the following text, we will discuss recent progress on ionomics studies of several major types of metabolic diseases. A summary of the main ionomics results obtained from the literature is given in Table 1.

**Table 1:** Major ionomics results for different types of metabolic diseases.

**Disease**	**Population (subgroup)**	**Number of subjects (case/control)**	**Sample type**	**Elevated elements**	**Decreased elements**	**Significant correlations (–, *p* < 0.05 and |correlation coefficient| > 0.3) or significantly changed ratios (/, *p* < 0.05) between elements in patients**	**Potential confounders adjusted or matched**	**References**
T2DM	Moscow, Russia (women)	93/1,236	Hair	Hg, K, Na	Ca, Co, Mg, Zn	–	–	(60)
T2DM	Guanajuato, Mexico	76/12	Serum	Al, Cd, Cu, Hg, Mn, Ni	Co, Cr, V	Mn–Cu, Mn–Zn, Se–Cu, Se–Mn, Se–Zn, Zn–Cu	Smoking status, other chronic diseases	(61)
		76/12	Urine	As, Cr, Cu, Zn	Cd, Co, Pb, Mn, Mo, Ni, Se	Se–As, Se–Cd, Se–Ni		
T2DM	Shanghai, China	122/854	Plasma	Cr, Cu, P, S, Se	Mg, Re	–	Age, sex, BMI	(62)
T2DM	Tanta, Egypt	40/36	Serum	Cu	As, Cr, Fe, Mg, Mn, Se, Zn	**As–Fe**^a^, ***Cu–Fe**, **Cu–Mg**, **Cu–Zn***, Fe–Mg, Se–Cr, Se–Fe, Zn–Fe, Zn–Mg	Age, sex, weight	(63)
T2DM	Suzhou, China	122/429	Plasma	As, Ba, Cd, Cr, Cs, Cu, Mn, Pd, Se, Sr, Zn, V	–	–	Age, sex, BMI, family history of diabetes, smoking status, drinking status	(64)
T2DM	Nord-Trøndelag county, Norway	128/755	Blood	Ag, Cd, Cr, Fe, Ni, Zn	Br	–	Age, sex, BMI, education, waist-to-hip ratio, income, smoking status, family history of diabetes, seafood intake, alcohol consumption	(65)
T2DM	Winston-Salem, USA	21/19	Toenail	Al, Ni, V, Zn	Cs	–	–	(66)
T2DM	Nanning, China	223/302	Serum	Ba, Ca, Fe, Se, Sr	V	–	Age, sex, BMI, smoking status, drinking status, physical activity	(67)
T2DM	Wuhan, China	94/94	Plasma	Al, Ba, Cu, Rb, Se, Sr, Ti, Tl, Zn	Mo	–	Age, sex, BMI, smoking status, drinking status, regular exercise, education, and family history of diabetes.	(68)
T2DM	Jinan, China	100/40	Blood	Co, Cr, Cu, Fe, Mn, Mo, V, Zn	Li	–	–	(69)
T2DM complication	Cartagena, Spain	31/43	Saliva	–	Co	–	–	(81)
		31/43	Plasma	Sr	–			
T2DM CVD complication	Wuhan, China	1,114/2,783	Plasma	As, Co, Cr, Mn, Mo, Ni, Sb, Sn, Sr, Ti, V, W	Cd, Cu, Pb, Se, Zn	–	Age, sex, BMI, education, smoking status, drinking status, physical activity, family history of CVD, hypertension, hyperlipidemia, baseline fasting plasma glucose, antidiabetic, duration of diabetes, eGFR	(82)
T2DM complication (microvascular)	Ankara, Turkey	118/40	Blood	–	Mg, Cr	–	–	(83)
T2DM	Sardinia region, Italy	68/59	Blood	–	Cr, Mn, Ni	–	–	(84)
T1DM	Sardinia region, Italy	192/59	Blood	–	Cr, Mn, Ni, Pb, Zn	–	–	
T1DM	Miami, USA	63/65	Serum	Cu, Mo	Mn, Se, Zn	Cu/Se, Cu/Zn	Age, sex, BMI	(85)
GDM	Padova, Italy	28/19	Placenta	Se	Cd	–	Age, birth weight, gestational week of GDM diagnosis, gestational week of delivery	(87)
GDM	Padova, Italy	36/36	Placenta	Hg, Si	Cd, Cu, Fe, Mn	–	Age, pre-pregnancy BMI, birth weight, gestational week of delivery	(88)
		35/30	Blood (maternal)	Cu	Al, Co, K, Rb, Sb			
		35/34	Umbilical cord blood	Al, Ca, Co, Cu, Mo, Na, Ti, Zn	Fe, K, Mn, Rb, Si			
GDM	Wuhan, China	241/1,849	Urine	As, Co, Ni, Sb, V	–	–	Fetal sex, pre-pregnancy BMI, smoking status, physical activity, maternal age, gravidity, occupational status, income, family history of diabetes	(89)
GDM	Yozgat, Turkey	60/52	Serum	Cd, Cu, Pb, Sb	Cr, Se, Zn	–	–	(90)
GDM	Beijing, China	335/343	Hair (maternal)	Hg, Sn	–	–	Age, pre-pregnancy BMI, education, occupation, parity, passive smoking, hair dye, hypertension during pregnancy, activity, folic acid supplementation	(91)
GDM	Wuhan, China	305/305	Plasma	Cu, Fe	Ca, Zn	–	Age, pre-pregnancy BMI, parity, income, education, passive smoking	(92)
Obesity	Moscow, Russia (women)	141/1,236	Hair	Hg, K	Ca, I, Mg, Zn	–	–	(60)
Obesity/overweight	Shanghai, China	516/460	Plasma	Cu, Fe, P, S, Sr	Mg	–	Age, sex, BMI	(62)
Obesity	Ankara, Turkey (children)	34/33	Serum	–	Co, V	–	Age, sex, pubertal stage	(103)
Obesity	Konya, Turkey (women)	45/50	Serum	Cu	Fe, Zn	–	Age	(104)
Obesity	Poznan, Poland (adolescents)	78/20	Serum	–	Ca, Mg, Zn	–	Age, sex	(105)
Obesity	Erzurum, Turkey (children)	85/24	Serum	Cu, Mn, Se	Zn	–	Age, sex	(106)
Obesity	Zagazig City, Egypt (children)	80/80	Serum	Cu	Fe, Se, Zn	–	Age, sex	(107)
Obesity	Makkah, Saudi Arabia	47/70	Hair	Cu, Se	Fe, Mn, Zn	Cu/Fe, Cu/Zn	–	(108)
Obesity	NHANES project, USA	1,127/5,475	Urine	Ba, Tl	Cd, Co, Pb	–	Age, sex, race/ethnicity, income, serum cotinine, television, video game, computer usage	(109)
Obesity	Reus, Spain (women)	42/51	Serum	As, Ba, Cu, Se, Sr	Ca, Fe, Mg, Na, Zn	–	Age	(110)
Obesity/overweight	Moscow, Russia	112/106	Hair	Al, As	Co, Cu, I, Mg, Mn, Ni, Zn	–	Age, smoking status, alcohol consumption, history of acute cardiovascular events, mineral supplementation, metal implants, occupational exposure to environmental toxins, environmental exposure to trace elements, acute inflammatory diseases	(111)
Obesity	Sonapur, Bangladesh	100/100	Serum	Na	Ca, Fe, K, Zn	–	Age, sex	(112)
Obesity (abdominal)/ MetS	Wuhan, China	816/576	Urine	Ba, Cu, Fe, U, Zn	–	Cu/Zn	Age, sex, BMI, smoking status, drinking status, income, education level, physical activity, family history of diabetes	(113)
Obesity	Dhaka, Bangladesh (women)	70/70	Serum	Na	Ca, Fe, K, Zn	Na–K	Age	(114)
Obesity	Yaroslavl, Russia	196/199	Serum	Se	Cr, V	–	Age, sex	(115)
			Hair	Cr, Se, V	Zn			
			Urine	V	–			
Obesity	Poznan, Poland (women)	40/40	Serum	Cu	Ca, Fe, Mg, Se, V, Zn	–	Age, BMI, and metabolic parameters	(116)
			Hair	–	Fe, Mn, V			
			Urine	Fe	Se, V			
Obesity	Kerman, Iran (children and adolescents)	48/38	Urine	As, Pb, Zn	–	–	Age, sex, BMI, physical activity, physical and clinical examinations	(117)
MetS	Shanghai, China	399/577	Plasma	Cr, Cu, P, S, Se, Sr	Mg	–	Age, sex, BMI	(62)
MetS	Seoul, Korea	161/295	Hair	As, Hg, K, Na, Pb	Ca, Mg, Zn	Ca/K, Ca/P, Na/Mg	Age, smoking status, alcohol use	(142)
MetS	Szczecin, Poland (aging men)	161/152	Serum	Zn	Mg	–	–	(143)
MetS	Beijing, China	80/65	Blood	Cd, Cu, Pb, Se	–	–	Age	(144)
MetS	Kaohsiung, Taiwan	826/1,618	Blood	Pb	–	–	Age, sex	(145)
			Urine	As, Cu, Ni	–			
MetS	China	628/1,481	Plasma	Ti	–	–	Age, sex, BMI, smoking status, drinking status, education, income, physical activity	(146)
			Urine	–	Fe, Se, V			
MetS	Ann Arbor, USA (women)	173/774	Urine	As, Co, Zn	–	–	Age, BMI, race, study site, education, smoking status, alcohol drinking, physical activity, total energy intake, menopausal status	(147)
MetS	Nanning, China (men)	254/1,716	Serum	As, Ba, Cd, Co, Cr, Cu, Fe, Mg, Mn, Ni, Pb, Rb, Se, Sn, Sr, V, Zn	–	–	Age, smoking, alcohol drinking, physical activity, education, family history	(148)
MetS	Taipei, Taiwan	40/110	Plasma	B, Cd, Cu, Li, Mg, Ni, Sr, Zn	–	–	Age, sex, BMI, smoking status	(149)
MetS	Shenzhen, China	149/1,128	Plasma	–	Mg, Mn	–	Age, sex, smoking status, drinking status, estimated glomerular filtration rate	(150)
NAFLD	Athens, Greece	119/70	Plasma	Cs, Fe, Tl	Ba, Zn	–	Age, sex, BMI	(180)
NAFLD	Nanning, China (men)	330/1,264	Serum	Ba, Ca, Fe, Mg, Mn, Ni, Pb, Sn, V, Zn	Mo	–	Age, marital status, education, smoking status, drinking status, MetS, insulin resistance	(181)
Hyperlipidemia	Konya, Turkey	46/37	Serum	Cd, Co, Ni	Cr, Fe, Mn, Mo, Se	–	Age, sex, blood pressure	(224)
Hyperuricemia	Shenzhen, China	344/1,062	Plasma	As, Cu, Mg, Se, Tl, Zn	Co	–	Age, sex, BMI, smoking status, drinking status, other diseases (hyperlipidemia, hypertension, DM, chronic kidney disease)	(247)
Hyperuricemia	USA	751/3,175	Urine	As, Cd	Co, I, Mn	–	Age, gender, BMI, smoking status, drinking status, income, education, fish/shellfish consumption, other diseases (hypertension, hypercholesterolemia, DM)	(248)
Osteoporosis	Banjarmasin, Indonesia (postmenopausal women)	15/23	Bone	As, B	Pd, Rb, Se	–	Age, history of previous disease	(267)
Osteoporosis	Izmir, Turkey (postmenopausal women)	491/237	Serum	-	Cu, Fe, Mg, Zn	–	Age, BMI, duration of menopause, family history of fractures, medical conditions, smoking status, medication use	(268)
Osteoporosis	Shanghai, China	37/14	Bone	–	Cu, Mn, Zn	–	Age, sex, BMI, serum albumin, biochemical blood indices, bone turnover markers	(269)
Osteomalacia	Rio de Janeiro, Brazil	8/603	Hair	Al, Ba, Ca, Cd, Fe, Mg, Mn, Sr	Hg	–	–	(278)
Rickets	Van, Turkey	30/30	Serum	–	Zn		Age, sex, medical history, physical examination	(279)

a Bold and italic: negative correlation.

### 3.1. Diabetes mellitus

DM comprises a group of metabolic diseases characterized by chronic hyperglycemia resulting from insufficient insulin secretion and/or insulin resistance in target tissues, which may lead to multi-organ dysfunction and failure (59). There are three main types of DM: type 1 DM (T1DM), T2DM, and gestational DM (GDM). T2DM accounts for more than 90% of all diabetic cases, which has become the most prevalent metabolic disease worldwide. It has been suggested that ion homeostasis may play an important role in T2DM and some other types of DM (3).

To date, the ionomes in blood (including whole blood, plasma, and serum), urine, hair, and even teardrops have been examined in different cohorts of patients with T2DM, implying that changes in elemental profiles represent a potential source of biomarkers of this complex disease (60–70). The majority of significantly changed elements in different biofluids (especially in the blood samples) are positively associated with T2DM, including several heavy metals (such as Cu and Cd) and Se whose accumulation in the body may be a high risk for T2DM. In contrast, concentrations of Zn and Mg in the blood and hair/nail samples were observed to generally have a negative association with the prevalence of T2DM. These trends are consistent with previous observations that environmental heavy metals and Se might affect insulin sensitivity and normal insulin regulatory function and cause pathoglycemia or even more serious illness such as T2DM, whereas deficiency of Zn, an essential trace metal for synthesis and secretion of insulin, may have a major impact on the pathogenesis of T2DM (3, 64, 67). Thus, such variations could be used for differentiating patients with T2DM from healthy controls. Moreover, significant correlations (|correlation coefficient| > 0.3 and *p* < 0.05) between elements and increased Cu/Zn ratio were also reported, suggesting possible benefits of the use of such information in detection and prognosis evaluation of T2DM (61, 63). By using machine learning algorithms [such as discriminate analysis, support vector machine (SVM), and random forest], several new approaches and models for the early detection of T2DM based on multi-elemental contents have been developed, which exhibit good prediction performance and may serve as a valuable tool of its diagnosis (66, 70–72). Very recently, the blood levels of several essential trace elements (such as V, Cr, Mn, and Se) were found to be negatively correlated with hemoglobin A1c (HbA1c) in diabetic subjects, especially in those with HbA1c ≥ 7.0%, suggesting mutual effects between them and metabolic abnormalities of blood glucose during the onset and progression of T2DM (69). On the other hand, although a significant number of studies only examined the concentrations of single or a small set of trace elements in biofluids of T2DM patients, similar or more complex patterns of changes were obtained for some of them in different regions of the world (73–80). For example, serum Cu, Zn, and Se levels were found to vary with the effect of glycemic control in T2DM patients, indicating a potential relationship between their homeostasis and the prognosis and treatment of T2DM (73). Long-term Ni and As exposures were also observed to be associated with elevated prevalence of T2DM in Chinese elderly and Mexican women, respectively, implying the harmfulness of more heavy metal contamination in the pathogenesis of T2DM (74, 75). Although González de Vega et al. reported lower plasma concentrations of total Se found in Spanish patients with T2DM which might be due to decreased levels of selenoprotein P (76), the majority of other studies on Se and DM suggest that Se may increase the risk of T2DM across a wide range of exposure levels (77). In addition, several recent ionomics studies have found that trace element imbalance is involved in the development of diabetic complications. Marín-Martínez et al. reported that decreased Co level in saliva and increased strontium (Sr) level in plasma were associated with the presence of chronic complications (both microvascular and macrovascular) in patients with T2DM, which provides some useful enlightenment for predicting diabetic complications (81). Other ionomics studies revealed that increased Sr and decreased Zn and Se levels in plasma have a significant association with incident cardiovascular disease (CVD) risk in patients with T2DM, whereas decreased Mg and Cr levels in blood might be involved in the progression of microvascular complications (such as diabetic retinopathy and diabetic nephropathy) (82, 83).

Ionomics studies have also been conducted for several other types of DM (84–87). One Italian group measured the blood levels of multiple trace elements in patients with T1DM and T2DM from Sardinia, and observed similar alternations of significantly changed elements between them (Cr, Mn, and Ni deficiency) in that area (84). Similar to T2DM, elevated serum level of Cu and lower levels of Zn and/or Mg were also found in subjects with T1DM from different regions, suggesting that perturbation in the balance of these essential metals may be related to the pathology of both types of DM (80, 85). Peruzzu et al. reported that whole blood levels of Cr and Cu were strongly correlated with glycemic control whereas Zn, Fe, and Se were associated with lipid metabolism in patients with T1DM, suggesting different effects of these elements on metabolic control in this disease (86). With regard to GDM, Roverso et al. carried out the first ionomics study of the placentas from women affected by GDM, and showed that a decreased level of Cd and increased level of Se might be linked to the molecular pathways of GDM (87). They further measured the ionomes of placenta, maternal whole blood, and umbilical cord blood samples from a larger cohort of GDM and normal pregnant women, which revealed that a number of elements detected in the umbilical cord blood of fetuses were significantly associated with the occurrence of GDM (such as increased Cu and decreased Fe levels) (88). This may indicate that elemental profiles in the cord blood provide more evidence for understanding the biochemical processes occurring during GDM. Moreover, several recent case-control and cohort studies as well as meta-analyses reported that exposure to toxic heavy metals [such as Ni, antimony (Sb), Cd, and Cu] and insufficient Zn content in the body in early pregnancy are associated with an increased risk of GDM, either individually or as a metal mixture (89–96). Overall, these studies demonstrate high complexity and tissue specificity of the ionomes in patients with different types of DM. However, elevated Cu and Se concentrations, decreased Zn concentrations, and increased Cu/Zn ratio in various biofluid samples might be one of the common risk factors for DM. Further effort is needed to investigate the molecular mechanisms underlying dyshomeostasis of trace elements in patients with different types of DM.

### 3.2. Obesity

Obesity is a chronic and complex disease characterized by excessive amounts of fat within adipocytes, which may result from insulin resistance, dysregulation in lipid and other metabolic balances, and inflammatory and/or hormonal processes (97). It may increase the risk of other diseases and health problems, such as T2DM, hypertension, CVD, and certain cancers. Both genetic susceptibility and environmental factors contribute significantly to the development of obesity (98). Among them, trace element and mineral dyshomeostasis has already been known to be associated with the pathogenesis of this disease (99). On the other hand, obese patients may be at a greater risk of developing imbalance of trace elements.

Before the use of ionomics in obesity research, many studies have shown a possible relationship between obesity and disturbance of homeostasis of certain trace elements, such as Zn or Fe deficiency and Cu overload, although the exact metabolic roles of these elements remain unclear (100–102). In the recent decade, a variety of studies have been carried out to examine the concentrations of more elements or ionomes in different biological samples of patients with obesity and healthy controls, which demonstrate more complicated changes linked to the prevalence of this disease (60, 62, 103–117). Consistent with previous observations, significantly increased levels of Cu and decreased levels of Zn and Fe in multiple samples (especially in serum) appear to be the most common features for obesity. However, the relationship between obesity and the circulating levels of Se is still controversial. A recent meta-analysis revealed that overweight/obese individuals might have lower levels of Se in the urine and nails but higher levels in the hair (18). Compared to DM, deficiency of essential trace elements may play a more important role in the pathogenesis of obesity and obesity-related metabolic dysfunction, especially in women and children. The Cu/Zn ratio was also mainly increased in several types of samples of the obese groups (in particular in diabetic obese individuals), and was negatively associated with increased risk of metabolic abnormalities in the urine (108, 113). In addition, exposure to environmental toxic metals in the body may significantly influence the ionome and other metabolic parameters of obesity. For instance, Skalny et al. reported that Hg-exposed overweight/obese subjects had quite different hair elemental profiles (such as elevated Se and Zn and decreased Mn and Mg levels) and a more adverse effect on metabolic parameters when compared to unexposed obese adults, suggesting that metabolic alterations observed in obesity may be partially related to Hg-associated disturbances in trace element metabolism (118).

A significant number of studies with larger cohorts have been performed recently to improve our understanding of the relationship between obesity and single or few essential/non-essential elements in various biofluids and tissues, which showed generally similar changes in the concentrations of some of them in different populations, such as increased serum and hepatic tissue Cu levels (119, 120), decreased serum, hair, and salivary Zn levels (121–123), decreased fingernail Se levels (124), and increased toxic heavy metal (such as Al, Hg, Pb, and As) contents (125–128). Many of these significantly changed elements are positively or negatively correlated with body mass index (BMI) and waist circumference, which are commonly used to estimate the risk of overweight, obesity, and some other metabolic diseases such as MetS (128, 129). Moreover, levels of some elements are associated with insulin resistance in obese adults. Kim and Song examined the concentrations of several trace elements in the hair of viscerally obese adults, and found that Cr and Se levels were inversely associated with insulin resistance, whereas Cu level has a positive correlation with insulin resistance (130). Thus, imbalance of these micronutrients might be involved in the development of obesity, which could be used as potential indicators for its incidence. Interestingly, although serum levels of Cu and Zn are thought to be significantly related to obesity in both children and adults, a recent study showed that they did not differ significantly between pre-obese and obese children, implying that alternations of the two metals are earlier than the occurrence of obesity (131). Nevertheless, further research is needed to confirm these findings prospectively in larger study populations and to extend the understanding of the roles of different elements in the prevalence of obesity.

### 3.3. Metabolic syndrome

MetS is a group of metabolic disorders, including abdominal obesity, hypertension, high blood sugar levels (insulin resistance or glucose intolerance), and dyslipidemia [high triglyceride and low high-density lipoprotein (HDL)-cholesterol levels]. It may raise the risk of CVD, T2DM, stroke, and other serious health problems (4). The cause of MetS has been intensively studied, and many factors (such as genetic and metabolic susceptibility) may contribute to the development of MetS (132). Among these, an imbalance in trace element status is an important risk factor and potential biomarkers for MetS (133). The important roles of several micronutrients (such as Fe, Zn, and Cr) in carbohydrate and lipid metabolism and their alterations in MetS have been discussed in multiple reviews, which provide evidence for the involvement of trace element dyshomeostasis in the pathology of MetS (4, 134–136).

In early years, a limited number of studies have been conducted to explore the distribution and relationships of selected trace elements in different samples of patients with MetS and normal controls, which revealed that some of these elements might be related to the incidence and course of MetS (137–140). For instance, elevated circulating Cu levels and body Fe stores as well as reduced serum levels of Zn were reported in patients with MetS in different areas. On the other hand, controversy exists with regard to the relationship between MetS and Se levels in body, probably due to differences in study design, methodology, and population characteristics (141).

Several ionomic profiling studies with large cohorts of patients were carried out in the recent decade (62, 142–150), which basically support previous observations that accumulations of Cu and Fe in the body are strongly associated with the increased prevalence of MetS. Moreover, imbalance of additional elements were detected in different biological samples of MetS cases, implying a high degree of variability of the ionomic patterns in patients with MetS. Generally, the majority of significantly changed elements have positive associations with MetS, especially environmental toxic metals (such as Pb, Cd, and As), implying that exposure to these elements affects the body composition and metabolic profiles and further exacerbates the risk of MetS (142, 144–149). In contrast, decreased Mg levels were often observed in the blood (including plasma and serum) and hair samples from patients from different regions (62, 142, 143, 150), which could influence glucose metabolism and insulin sensitivity and action as well as promote chronic inflammation, linking Mg deficiency with developing MetS and other metabolic disorders such as T2DM and obesity (151). So far the ionomic information available for assessing the potential relationship between Zn status and the incidence of MetS is inconsistent and may vary by gender. It seems that increased Zn levels in the serum/plasma samples may lead to the occurrence of MetS in men but have a protective effect in women (143, 148, 152). In addition, some of the elements were found to be significantly associated with different MetS components. For example, Wen et al. have shown that urine Cu and blood Pb levels were positively associated with obesity-related indices such as body roundness index and body/visceral adiposity index, and urine Ni was positively correlated with lipid accumulation product in a large cohort of MetS cases (145). Increased Ca/Mg ratio in the hair was also found to have a positive correlation with insulin resistance in Korean adult males (153). Very recently, Zhang et al. investigated the relationship between the blood concentrations of several essential metals and MetS components in Chinese children, which revealed that different metals correlate with MetS components individually, such as Cu (positively associated with elevated waist and triglyceride levels) and Mg (positively associated with reduced HDL-cholesterol levels) (154). Such information may suggest a potential use of ionomic data in monitoring the progression of MetS components and helping determine the proper treatment strategies.

Besides the ionome-level studies mentioned above, more case-control studies and meta-analyses recently focused on single or several trace elements and explored their differences between MetS patients and healthy controls in different biological samples (17, 155–164). Although some of these results appear to be contradictory and ambiguous, it appears that elevations of Se, Cu, Zn (in males), and toxic metals (such as Cd and Hg) and reduction of Mg in blood samples are associated with increased risk of MetS and its components. Besides Zn, gender difference may affect the correlation between MetS and some other elements such as Se (165). Moreover, interactions between different elements may modify their own roles in MetS, e.g., the harmful effects of Hg exposure on MetS could be significantly attenuated by high levels of Se in toenails (166). Future studies should aim to unravel the mechanisms underlying the associations between these elements and the development of MetS to assist in the prevention and early intervention of this disease and its components.

### 3.4. Non-alcoholic fatty liver disease

NAFLD is the most common chronic liver disease worldwide, which is characterized by excessive fat accumulation (≥5%) in hepatocytes in the absence of excessive alcohol consumption (167). It encompasses a broad histological spectrum ranging from simple hepatic steatosis to non-alcoholic steatohepatitis (NASH), cirrhosis, and hepatocellular carcinoma (HCC) that may develop further (168). Because of the lack of efficient diagnostic methods and therapeutic targets, early detection and treatment of NAFLD still face many challenges. The underlying mechanism for the pathogenesis and progression of NAFLD is complex, and disturbance in trace element and mineral metabolism and interactions have been identified as one of the key risk factors of this disease (169, 170). As NAFLD is closely associated with the components of MetS, obesity, and T2DM, a change in nomenclature from NAFLD to metabolic-associated fatty liver disease (MAFLD) has been recently proposed, which may better reflect the underlying pathophysiology of this disease (171). Here, we still use NAFLD in the following sections.

The association between certain trace elements and NAFLD risk has been reported since 1990s, such as hepatic Fe overload and lower liver and serum Cu concentrations in different types of NAFLD patients (including pronounced hepatic steatosis, NASH, and even NASH-related cirrhosis with HCC) (172–177). The serum ferritin, a marker of Fe storage, has been considered as an independent predictor of histologic severity and fibrosis in patients with NAFLD, which may also indicate that changes in Fe speciation [say, the conversion between Fe(II) and Fe(III) species] are related to the pathogenesis of this disease (178). However, children with NASH exhibit decreased serum Fe levels with no significant Fe accumulation in the liver, implying a new mechanism for Fe deficiency in pediatric NASH (179). To date, very few ionomics studies have been carried out for NAFLD, which mainly used blood samples from patients. For the first time, Asprouli et al. measured plasma ionomes in different stages (mild, moderate, and severe) of NAFLD subjects in Greece, which demonstrated that Zn had a negative association with the severity of the disease whereas cesium (Cs) showed a positive correlation (180). Li et al. examined the relationship between NAFLD and serum contents of a variety of metals based on cross-sectional and longitudinal analyses in Chinese men, and found that depressed Mo and elevated Zn levels were associated with an increased risk of NAFLD (181). It should be admitted that ionomics in NAFLD is still at the very beginning of its development. So far ionomic profiling of other biological samples in NAFLD patients is not yet available.

In the last few years, a large number of studies have explored the correlation between single or several elements and the development of NAFLD. First, consistent with previous findings, Fe accumulation appears to be the most significant and common marker for NAFLD, and indicators of Fe status (e.g., serum Fe and ferritin levels) could be used for the prediction of disease severity and adverse outcomes (such as the risk of advanced hepatic fibrosis and HCC) in different populations (182–186). Second, decreased Cu and Mn concentrations in hepatic tissues, serum, and/or hair were reported in different cohorts of NAFLD patients, and higher Cu or Mn levels achieved significant protective effect against NAFLD, especially in men (187–192). In addition, recent Cu isotopic composition analysis showed that serum 65Cu/63Cu ratio was significantly lower in NAFLD patients and remained stable during disease progression, suggesting its potential for early diagnosis of NAFLD (193). Third, although the relationship between Zn deficiency and the incidence of NAFLD is still uncertain, low serum levels of Zn have been proposed to serve as an independent risk factor for poor prognosis in patients with NAFLD (194–197). In contrast, the roles of Se in different types of NAFLD are complex and conflicting (198). It seems that increased plasma/serum Se levels are positively associated with elevated prevalence of liver steatosis but negatively associated with that of hepatic fibrosis or HCC (198–201). Finally, exposure to toxic heavy metals (such as Pb, Cd, Hg, and Ni) was significantly associated with the onset and progression of NAFLD in different regions of the world, which is probably a causative factor of hepatic steatosis and fibrosis in some patients (202–208). Moreover, increased serum Ca and phosphorus (P) levels were also reported as independent risk factors for fatty liver in Korean population (209). Although not at the ionomic level, these single/few element-based studies might still provide useful information regarding the changes of trace elements in biological samples of NAFLD patients. Further investigation is necessary to evaluate the value of ionomic changes in the early detection of NAFLD individuals.

### 3.5. Hyperlipidemia and hyperuricemia

Hyperlipidemia (also referred to dyslipidemia) represents the condition of abnormally elevated levels of one or more lipids and/or lipoproteins in the blood, mainly including high levels of total cholesterol (hypercholesterolemia), triglycerides (hypertriglyceridemia), and low-density lipoprotein (LDL)-cholesterol or low levels of HDL-cholesterol. It is a significant risk factor for a variety of diseases such as fatty liver, CVD, DM, neurodegenerative diseases, and acute pancreatitis (210). Hyperuricemia (serum uric acid level ≥6.8 mg/dL), a key risk factor for the development of gout, is caused by increasing production and/or decreasing clearance of uric acid (the end product of purine metabolism) (211). In addition, hyperuricemia may also increase the risk of chronic kidney disease, CVD, metabolic disorders such as hyperlipidemia, obesity, and other insulin resistance-related syndromes (212). In recent years, the incidence of hyperlipidemia and hyperuricemia has increased significantly, which have become two of the most common health threatening disorders worldwide. Therefore, the prevention and treatment of the two pathological conditions are important for a reduced risk of more severe outcomes such as atherosclerosis, coronary heart disease, and renal disease (213, 214). It has been proposed that trace elements and minerals play an important role in the regulation of lipid and purine metabolism. Thus, investigation of the association between trace element imbalance and the occurrence of hyperlipidemia and hyperuricemia might be helpful to study the etiology and pathogenesis of the two metabolic diseases and to develop new lipid- and uric acid-lowering drugs.

The relationship between hyperlipidemia and trace element homeostasis has been analyzed in early times. Deficiency of several essential elements (Zn, Fe, Cr, and Se), Cu excess, and significant urinary excretion of Fe, Zn, and Cu were observed to be associated with the prevalence of hyperlipidemia (215–219). However, Cu deficiency and hepatic Fe overload were reported to contribute to hypertriglyceridemia and hypercholesterolemia in rats, and the genetic mechanisms driven to hypertriglyceridemia might favor Fe deposits (220–222). Moreover, some lipid-lowering agents (e.g., statins) could greatly influence the serum levels of multiple trace elements such as Zn and Cu (223). In the last decade, very few ionomics studies have been conducted for hyperlipidemia patients. Yerlikaya et al. found that lower levels of essential elements (Cr, Fe, Mn, Mo, and Se) as well as higher levels of Co, Ni, and Cd in serum are associated with an increased risk of primary hyperlipidemia, suggesting an important role of these elements in the regulation of lipid homeostasis (224). On the other hand, several studies have either confirmed previous observations or revealed additional elements that are associated with hyperlipidemia. For example, high Mn exposure could decrease the risk of high triglycerides (225), while elevated blood/serum levels of toxic heavy metals (such as Hg, As, and Pb) may increase the risk of different types of hyperlipidemia (226–228). Very recently, Barragán et al. examined the associations of plasma essential trace elements with hyperlipidemia in a Mediterranean population, which demonstrated that plasma Mg concentrations and combined effect of plasma Zn, Cu, and Se levels had a positive association with hypercholesterolemia whereas plasma Mn concentrations would have negative effects on the amount of plasma lipids (229, 230). A U-shaped link was suggested to exist between plasma Se levels and hypercholesterolemia, indicating that both low and high Se concentrations might have negative effects on plasma lipids (230).

Dyshomeostasis of trace elements has also been implicated in the incidence and progression of hyperuricemia for a long time. In general, circulating levels of several essential metals (such as Ca, Fe, and Cu) and toxic metals (Pb and As) showed positive correlations with the prevalence of hyperuricemia in different populations (231–238). In contrast, concentrations of Mg in serum and I in urine were inversely associated with the prevalence of hyperuricemia (239, 240). Although the relationship between Zn levels and hyperuricemia has not been explored, dietary Zn intake was reported to be inversely correlated with hyperuricemia in different regions, suggesting that Zn deficiency might increase the susceptibility of hyperuricemia (241, 242). Interestingly, there was a discrepancy of the association between blood Cd, serum uric acid levels, and hyperuricemia in different genders. Several studies have shown that blood Cd levels had a positive correlation with increasing risk of hyperuricemia in men (243, 244) or a negative correlation in women in Chinese and Korean populations (237). However, an opposite pattern was observed among populations in the U.S., in which serum Cd levels (in the acceptable range) were positively associated with increased prevalence of hyperuricemia in women, but inversely associated in men (245). This may suggest that different compositions of blood, race, and different ranges of blood Cd can affect the effect of Cd exposure on uric acid levels (246). So far the ionomics study on hyperuricemia is almost a blank. Wang et al. measured the plasma levels of 13 metals in hyperuricemia patients and controls in Chinese Han adults and found that higher plasma levels of several metal ions (particularly Zn and As) and lower levels of Co might increase hyperuricemia risk (247). Ma et al. reported that increased urinary levels of toxic metals (As and Cd) and decreased levels of essential metals (Co, Mn, and I) might have a positive combined effect on hyperuricemia (248). A systematic meta-analysis has been recently conducted to clarify the relationship between exposure to certain metals and the risk of hyperuricemia, which revealed that exposure to As, Ca, Cd, and Pb is associated with an increased risk of hyperuricemia whereas Mo exposure appears to be associated with a decreased prevalence of this disease (249). All these findings reinforce the importance of heavy metal (especially environmental toxic metal) accumulations in the risk of hyperlipidemia and hyperuricemia in general population. Moreover, alternations specific for each of the two metabolic diseases (e.g., elevated serum Co levels in hyperlipidemia and decreased plasma Co and urinary I levels in hyperuricemia) might help to provide potential markers for distinguishing them before clinical symptoms appear. Further ionome-level studies are required to validate the above results and to explore new patterns of elemental profiles in more biological samples and larger cohorts.

### 3.6. Osteoporosis and osteomalacia

Osteoporosis is a systemic bone metabolic disease characterized by bone mass reduction and bone microstructure destruction, which increases the risk of fracture, particularly in older postmenopausal women. The pathogenesis of osteoporosis is complex and involves the interaction of genetic, hormonal, environmental, and nutritional factors (250). Osteomalacia (or rickets in children) is a metabolic bone disorder of decreased mineralization of newly formed bone due to the lack of available Ca, P, or vitamin D. The major causative factors of osteomalacia include nutritional deficiency, impaired absorptive capabilities, and renal insufficiency or dialysis (251). Trace elements have been known to be essential for maintaining bone health, whose imbalance may be significantly associated with the disorders of bone metabolism and further lead to osteoporosis and/or osteomalacia (252, 253). Analysis of the relationship between trace element contents and osteoporosis/osteomalacia may assist in the prevention, diagnosis, and treatment of bone metabolic diseases.

The correlation between certain trace element and mineral levels within the body and osteoporosis has been examined in a large number of population-based studies, case-control cohort studies, and meta-analyses since the early 1970s. In general, reduced circulating levels and/or elevated urinary excretion of a variety of essential metals [such as Ca, Cu, Zn, Mg, Se, and boron (B)] as well as accumulation of Fe, Mn, and toxic heavy metals (Cd, Pb, and As) are strongly related to the prevalence of osteoporosis (254–266). In the recent decade, with the widespread use of ionomics approaches in various complex diseases, several studies have been carried out to provide evidence of the relationship between trace elements in blood and bone tissues and osteoporosis. For the first time, Noor et al. analyzed the difference of the bone concentrations of 27 elements from Indonesian postmenopausal women with and without osteoporosis, which showed that increased B and As and decreased Se levels in bone tissues might directly contribute to microarchitectural abnormalities in osteoporotic bones (267). Another ionomics study suggested that low serum levels of Cu, Zn, Fe, and Mg are associated with the incidence of osteoporosis in Turkish postmenopausal women, implying that Fe deficiency is also one of the risk factors for the development of osteoporosis (268). Lin et al. measured multi-elemental contents in human bone tissues of older Chinese adults and found a significant correlation between decreased Cu, Zn, and Mn levels and increased risk of osteoporosis, indicating that both Mn exposure and deficiency (although very rare in human body) might lead to abnormal bone metabolism, osteoporosis, and even fractures (269). A SVM-based prediction model for bone mineral density loss and osteoporosis was recently proposed by using urinary concentrations of several toxic metals (such as As and Cd) and some other features, suggesting the possibility to develop new methods for the early detection of osteoporosis based on elemental distribution in biofluids (270). Additionally, lowering excessive toxic metals and/or supplementation of deficient essential elements might be valuable for the therapy of osteoporosis.

In the past several decades, besides low serum levels of Ca and P that have been proposed as a screening test in patients who have clinical symptoms suggestive of osteomalacia (271), elevated concentrations of several other elements have been observed in different biological samples of osteomalacia patients, such as Al, Fe, Cd, and Sr in bone or serum and Pb in blood or hair (childhood rickets) (272–275). In contrast, Fe deficiency was reported in autosomal dominant hypophosphatemic rickets, a rare form of rickets caused by mutations in the fibroblast growth factor 23 (FGF23) gene, implying that monitoring the Fe status is helpful for diagnosis, prognosis, and treatment of this genetic disease (276). Moreover, Ca/Zn ratio in bone was found to be related to the degree of osteomalacia in Cd-exposed subjects, indicating that interactions among these metals might be involved in the pathogenesis of osteomalacia (277). To date, ionomics studies on osteomalacia/rickets are extremely rare. Previously, Miekeley et al. reported that elevated concentrations of Ca, Cd, Al, Fe, and several other elements in hair were associated with the incidence of osteomalacia, which could be used as a complementary tool for its early detection (278). Dogan et al. examined multiple trace element levels in serum and found significantly decreased Zn concentrations in children with nutritional rickets associated with vitamin D deficiency, suggesting an important role of Zn in the early steps of bone maturation (279). However, the correlations between many other trace elements and the prevalence of osteomalacia/rickets are still unclear. Future investigations are necessary to identify more ionome-level variations for bone metabolic diseases.

## 4. Development of integrated ionomic fingerprints for metabolic disease

**Figure 1:**
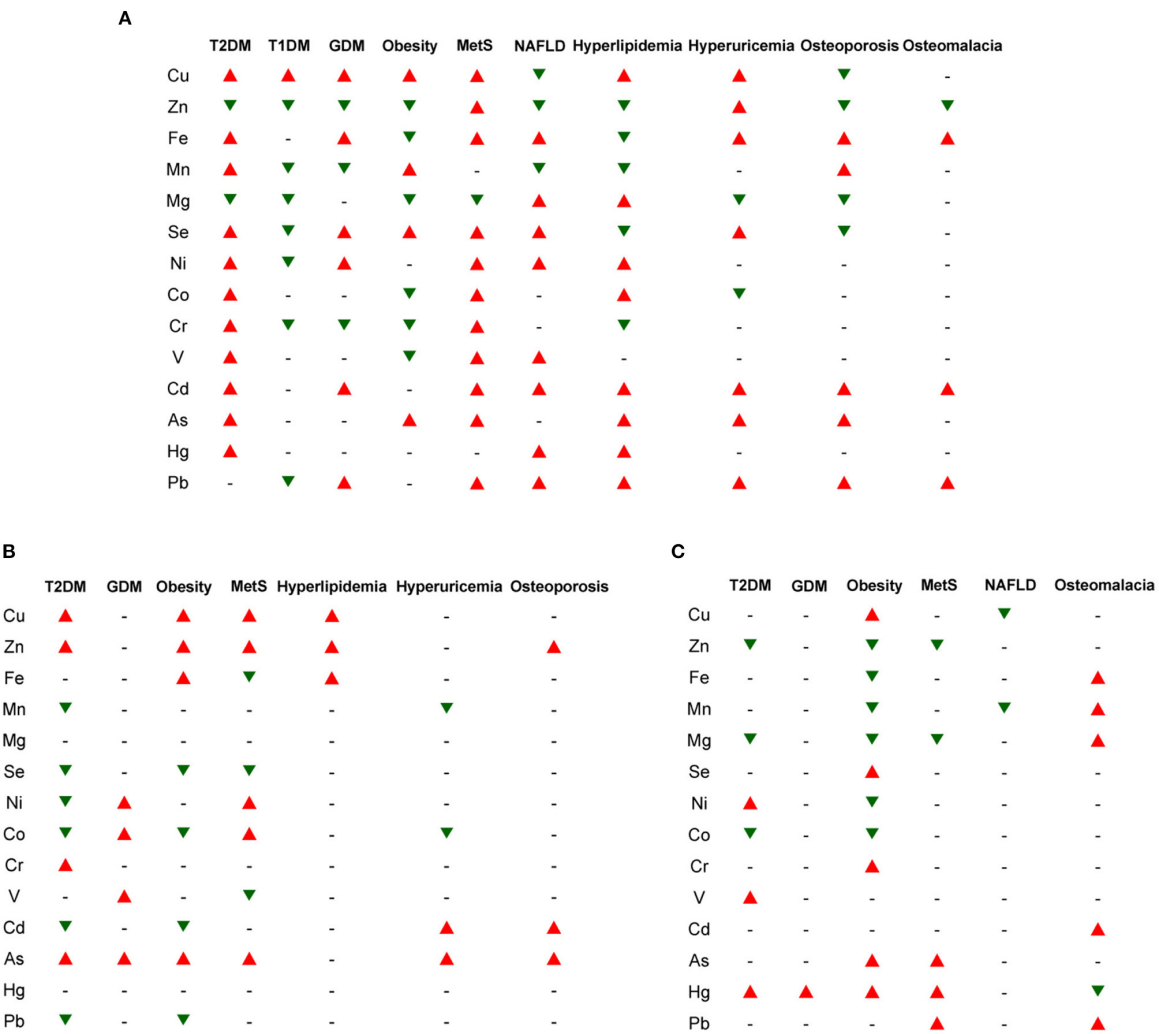
The general trends in the changes of major elemental concentrations in different biological samples for several types of metabolic diseases. **(A)** blood (including whole blood, serum, and plasma); **(B)** urine; **(C)** hair/nails. The red and green triangles indicate increased and decreased trends of elemental levels, respectively, which have been reported in the majority of ionomic and other related studies using the corresponding samples from patients. The short line (–) represents unclear or no significant change. T1DM, type 1 diabetes mellitus; T2DM, type 2 diabetes mellitus; GDM, gestational diabetes mellitus; MetS, metabolic syndrome; NAFLD, non-alcoholic fatty liver disease.

Comparison of the integrated ionomic profiles in different biological samples for major types of metabolic diseases has demonstrated common/specific trends in the changes of elemental contents (Figure 1). Considering that the number of ionomics studies for each subtype of blood samples (i.e., whole blood, serum, and plasma) is very limited for most metabolic diseases, in this review, we combined different subtypes of blood samples to try to give a general trend of the changes of elemental concentrations in the blood as previously adopted for cancer blood metallomics (280).

Among all examined ions, Cu levels are significantly increased in almost all examined samples (except hair/nails) from patients with various metabolic diseases (except NAFLD and bone metabolic diseases), indicating that Cu accumulation is one of the common risk factors for a wide variety of metabolic diseases and might act as a general marker for diagnosis and poor prognosis of them. Elevated concentrations of Se and environmental heavy metals (such as Ni, Cd, As, and Pb) and reduced levels of Zn and Mg are often observed in the blood/serum/plasma and hair/nails in patients with metabolic diseases, suggesting that alternations in the homeostasis of these elements may also serve as indicators of these diseases. In contrast, higher excretion of Zn and lower excretion of Se, Cd, and Pb in the urine were observed in several major types of metabolic diseases (especially T2DM, obesity, and MetS), which may partially contribute to the imbalance of these elements within the patients. In addition, the Cu/Zn ratio is also increased in the serum/hair from patients with T2DM and obesity, indicating its potential as a general circulating marker for these diseases.

**Figure 2:**
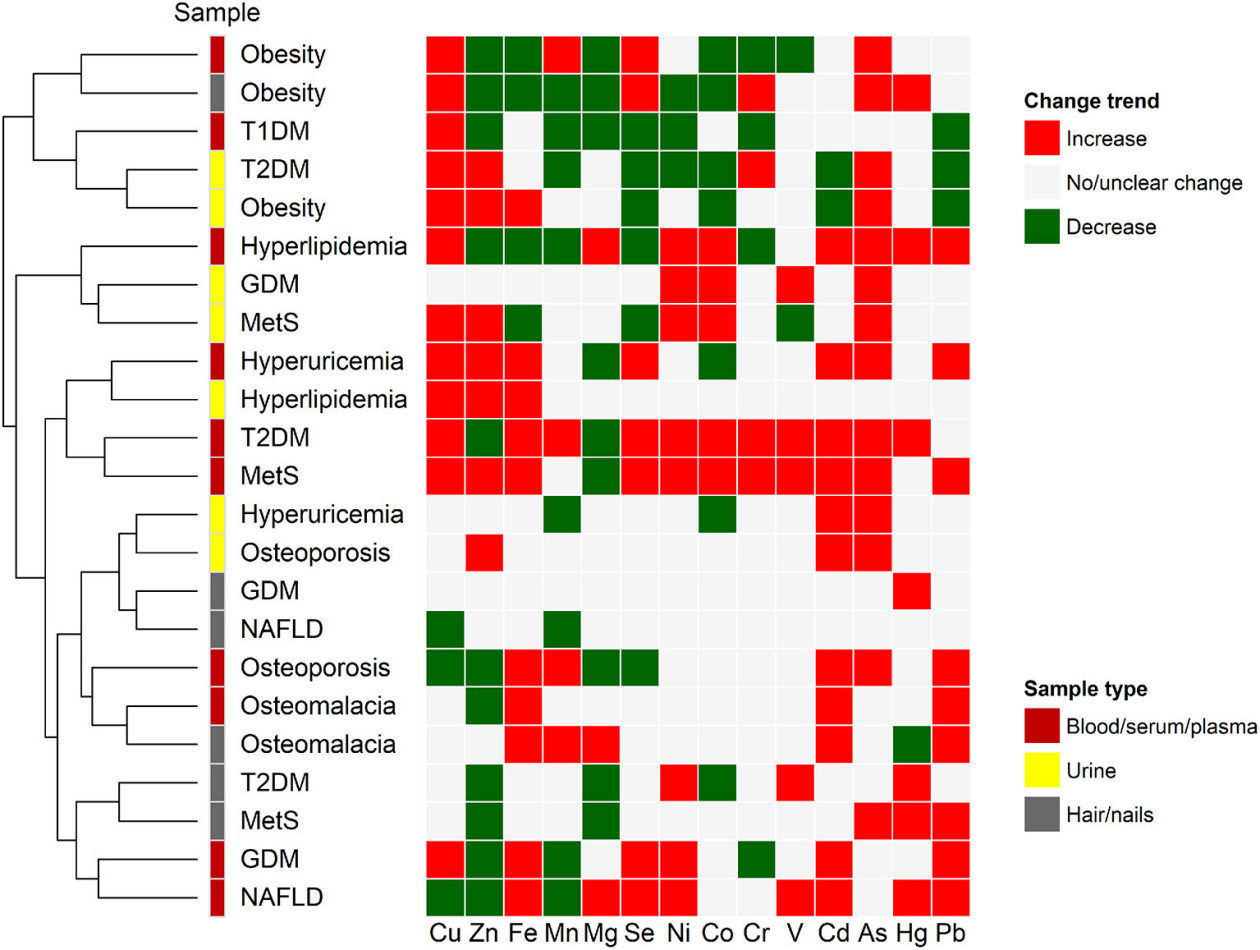
Clustering of ionomic profiles for metabolic disease. Alternations in the concentrations of major elements in several types of biological samples of patients with different metabolic diseases were analyzed using hierarchical clustering approach. Red and green colors represent increased and decreased trends of elemental concentrations, respectively, when compared with healthy controls. The gray color represents no significant or unclear change. Abbreviations of different diseases are the same as those in Figure 1.

Although the elemental contents are distinct and highly dynamic in different samples from patients with same/different metabolic diseases, each sample type appears to have specific ionomic pattern, which could be further used to develop novel biomarkers or prediction models for the respective disease. Clustering analysis revealed that same sample types from patients with different metabolic diseases may have quite similar or correlated ionomic patterns (Figure 2), such as blood (including whole blood, serum, and/or plasma) and hair/nails from T2DM and MetS as well as urine samples from T2DM and obesity, which might be associated with common pathogenetic mechanisms (say, insulin resistance) of these diseases (281). On the other hand, although most sample types from same/different diseases showed diverse elemental profiles, certain samples from patients with the same disease were clustered together, suggesting a potential relationship between them (Figure 2). For example, blood and hair/nails from obesity patients have same trends in the changes of major elemental concentrations (say, decreased levels of Zn, Fe, and Mg and increased levels of Se), whereas the urine samples of obesity have somewhat different elemental patterns (such as increased Zn and Fe, decreased Se and Cd, and unchanged Mg levels). Previous human biomonitoring programs have shown significantly different elemental contents between hair and urine samples in physiological conditions (282). It has been suggested that urine can be used for the monitoring of very low levels of elements and the assessment of recent exposure of heavy metals due to its rapid clearing and metabolism, whereas hair and nail samples may serve as indicators for long term exposure of environmental chemicals and toxic metal accumulation in various populations (282–284). Moreover, the elemental ratio or correlation analysis (shown in Table 1) may offer additional clues with respect to the complex crosstalk between elements in different types of metabolic diseases.

Although ionomics provides valuable insights into the mineral and trace element composition in a living organism and their relationship with different physiological and pathological conditions, there are still limitations for its potential role in diagnosis and prognosis of various diseases including metabolic diseases. One major limitation of current ionomics studies is the relatively small number and the varying characteristics (e.g., age, gender, and history of smoking and drinking) of patients and controls enrolled in different projects, which might restrict the power of ionomic evaluation when considering the high variability of trace element contents in biological samples. Another limitation is that ionomics study could not provide a definite cause and effect relationship between dyshomeostasis of trace elements and the development of metabolic diseases. Moreover, studies on the connection between alternations of elemental profiles and the levels of element-binding proteins (say, metalloproteins) and the dynamic interplay between elements are still lacking for most metabolic diseases. Nevertheless, ionomic analyses of biofluids, hair, nails, and other related tissues may be useful to give a comprehensive overview of the complex interactions between trace elements and minerals and metabolic diseases and help to generate new fingerprints for diagnosis, prognosis, and even for evaluation of therapeutic efficacy in these diseases, which cannot be solved only by the assessment of single or very few element levels.

## 5. Conclusions and perspectives

Disruption of ion metabolism and homeostasis plays a significant role in various diseases including cancer, metabolic diseases, neurological diseases, CVD, and many more. In the recent decade, ionomics has become a powerful tool for systematically investigating the composition and distribution of trace elements and minerals in different biological systems. However, our understanding of the relationship between ionome and the pathogenesis of metabolic diseases is limited so far.

This review focuses on several major types of metabolic diseases and introduces recent progress in the application of ionomics in the study of these diseases, which may raise the possibility of using such information for early detection and prognosis of patients with metabolic disease. Increased Cu concentrations in the majority of biological samples appear to be the most significant marker for various metabolic diseases. Elevated circulating levels of Se and environmental toxic metals and reduced Zn levels have also been demonstrated to be the common risk factors for the majority of metabolic diseases. Other elemental and inter-elemental biomarkers may also reflect the imbalance of ion contents that occurs in different samples of same/different types of metabolic diseases. In spite that the use of ionomics in the field of human disease remains at the initial stage, it may help to depict the crosstalk among different ions in disease pathogenesis and provide a potential source of diagnostic, prognostic, and predictive biomarkers, particularly in the context of metabolic disease. Future research with larger and more representative samples and/or more complex elemental composition (including chemical speciation and isotope analyses) is encouraged to improve our current understanding of the association between the ionomic network and the onset and progression of different types/subtypes of metabolic diseases.

## Author contributions

YZ and HY conceived and wrote the manuscript. BH, JJ, and YX reviewed and edited the manuscript. All authors contributed to the article and approved the submitted version.
